# Using a human security lens to examine experiences of violence against women in long-term encampment

**DOI:** 10.1371/journal.pone.0336028

**Published:** 2025-11-06

**Authors:** Njeri Kagotho, Njoki Maina Gitau, Gabriel Lubale, Niklas Mayer

**Affiliations:** 1 Ohio State University, College of Social Work, Columbus, Ohio, United States of America; 2 Kenya Institute of Migration Studies, Population Studies and Research Institute (PSRI), University of Nairobi, Nairobi, Kenya; 3 Maastricht University, Maastricht, Netherlands; Universidad Católica Sedes Sapientiae: Universidad Catolica Sedes Sapientiae, PERU

## Abstract

**Background:**

Issues of violence in humanitarian migration are complex, and more so when individuals live in long-term encampment. A gendered view of violence and migration brings into sharper focus the protracted situation for female humanitarian migrants and the specific vulnerabilities to which they are exposed when forced to migrate. Informed by the human security paradigm, this study explores women’s experiences with violence in Kakuma Refugee Camp, Kenya.

**Methods:**

Convenience sampling methods were used to enroll women of Somali origin (*N* = 260). A structured questionnaire consisting of culturally and linguistically validated measures was administered by trained community health promoters. Structural equation modeling was employed to examine the relationship between variables, allowing for an analysis of the factors influencing experiences of violence for women in the camp.

**Results:**

The average length of stay was approximately14 years. Findings indicate that individual and household-level factors—marital status, age, and household food security—were associated with experiences of violence. Further, sociocultural and security-level factors—attitudes toward intimate partner violence, access to security services, and immigration services—were associated with experiences of violence.

**Conclusion:**

This study contributes to the existing and growing body of literature on the determinants of violence against female humanitarian migrants in long-term encampment and their lived experiences. Policy recommendations are provided.

## Introduction

Issues of violence in humanitarian migration are complex, particularly when individuals live in long-term encampment. The lack of a nuanced gendered lens in navigating the complexities of violence and humanitarian migration, especially in the context of conflict and forced mobility, has led to generalized and simplistic policy formulation and recommendations that are barely gender responsive [1]. It thus behooves scholars and policy makers to examine with a critical lens violence in the context of humanitarian migration [1,2].

Informed by the human security paradigm [3,4], this study examines women’s experiences with violence once they reach a destination country. Unlike other security frameworks, the human security paradigm recognizes that security extends beyond the state and into other interconnected, humancentric dimensions. This strengths-based approach emphasizes preventive factors by first identifying and then addressing the causes of human insecurity. Further, we broaden our understanding of security issues through a gendered lens, an approach which intertwines women’s daily lives with larger structural migration dynamics [4,5]. Violence profoundly affects gender roles and identities as it can reinforce harmful gender norms and impact individuals’ ability to participate fully in society. This nuanced perspective, which integrates the human security paradigm and a gender lens, enhances our understanding of and ability to address the diverse experiences and challenges individuals face—particularly in relation to security.

Specifically, the present study aims to examine the factors associated with experiences of violence post-migration among Somali women in long-term encampment in the Kakuma Refugee Camp. For more than three decades, Kenya has remained a significant host country for refugees, with the Kakuma camp hosting approximately 288,206 humanitarian migrants [6]. Protracted stays in refugee camps—lasting 5 years or more—are particularly important to study because they can profoundly affect the social, economic, and psychological well-being of residents [7].

## Literature review

### Migration and violence

The conventional understanding of violence and humanitarian migration is that migrants flee persecution and violent situations (often political) to seek peace and refuge in another country. And while this is true, it remains at best a rather simplistic correlation that limits cognizance of the full scope of the violence–migration nexus [8]. Research demonstrates that host communities and governments can be conduits of systemic oppression of humanitarian migrants. The sometimes problematic and narrowly framed view of migration as a “security threat” has seemingly inoculated receiving states against reprimand when they choose to mete out “harsh and inhumane” actions against migrants. It is not uncommon for tropes used in the “othering” of migrants to tie into “securitization” of these groups and thus to supposedly justify extreme measures meted out against migrating populations [9,10]. There is a salient gap between the policies, conventions and protocols put in place to address post-migration violence and their implementation. Governments sometimes obfuscates this gap by treating migrants as security threats and choosing to prioritize territorial integrity over human rights [10]. The reclassification of humanitarian migrants, and especially those from Somalia, into “security threats” in Kenya has ushered in changes to policy surrounding the registration and allocation of immigrant status of refugees and the issuance of registration documents and travel documents [9].

### Women and violence

Gender is understood as a complex set of socially constructed expectations and norms that shape individuals’ identities and behaviors. Regressive informal institutions (unwritten patterns of social behavior that are communally enforced) often place men in positions of power over women and girls [11,12]. These institutions dictate the norms and mores associated with women’s agency and vulnerability. This includes attitudes that shape familial and interpersonal relationships, characterized by both overt and covert tactics of abuse, encroachment on bodily integrity through early child marriage, and female circumcision [13,14]. For instance, preconceived gender norms and gender socialization advance gender-based violence, including intimate partner violence (IPV) [2]. The World Health Organization estimates that 33% of women in Africa experience IPV in their lifetime [15]. Even in instances when comprehensive security and justice apparatus are in place, cultural taboos around victim blaming and rigid gender norms can prevent survivors from accessing these services [16]. The psychological consequences of these gendered experiences impede a woman’s ability to fully engage in daily activities.

In the context of conflict, gendered violence has become an area of increased concern and focus, highlighting the protracted situation and specific vulnerabilities faced by female humanitarian migrants when forced to [1,4]. Humanitarian migrants are especially susceptible to violence, given their transient existence. Civil war, human rights infringements, various forms of state fragility, government insurgencies, threats of political terror attacks, genocides, repressions, and political regime transitions positively correlate to increased migratory flows [17]. For migrant women, violence occurs within the intersecting context of gender and sociocultural and policy concerns. In addition to the state violence directed toward humanitarian migrants, research shows that within the confines of encampment, humanitarian migrants are further exposed to repeated emotional and physical violence by the camp administrators or by fellow migrants [18]. The literature identifies sexual violence, physical safety concerns, ineffective law enforcement, weak internal security, and lawlessness as specific issues faced by women during encampment [19,20].

### Human security paradigm

To effectively integrate the themes of women’s violence and migration, this multidisciplinary study considers a framework that address both structural and individual factors. The conventional models of migration security focus on the importance of the supreme state and its potential to maintain or disrupt security. In doing so, focus on the security of the individual is slighted [21,22], as these national security frameworks tend to securitize migration issues, often viewing migration through the lens of potential threats to national sovereignty and public safety. In response to this lack of focus on humanitarian aspects of migration, the human security model emerged in the 1990s. In essence, human security is the avoidance of potential future risks and the conscious effort to prevent human beings from falling below a particular threshold of deprivation [3].

This paradigm defines human security as “freedom from fear and freedom from want” [3]. Freedom from want tackles liberation from chronic threats such as environmental disasters, diseases, hunger, and famine, while freedom from fear tackles the safety of the individual and protection from threats and violence meted out at a personal level or through state-sponsored violence. It is protecting human beings from severe and pervasive existential threats that include but may not be limited to domestic violence, drug trafficking, and terrorism [21]. Human security is premised on strengthening the resilience of communities and providing tenable solutions as a more effective means of combating peril. This paradigm fortifies communities to transition from humanitarian crises to long-term sustainable solutions, and strengthens the tenacity of communities in the face of natural and climate-related disasters [23]. It emphasizes the need to address the root causes of insecurity that disproportionately affect women, such as poverty, discrimination, and lack of access to resources [5].

Despite these strengths, feminist scholars have critiqued the paradigm’s gender neutrality [4]. To address this gap, it is essential to integrate a gender lens that examines how security threats and vulnerabilities are experienced differently across gender identities. And it is precisely because the paradigm offers the opportunity to contextualize power and intersectional identities, that scholars can address the gender-specific experiences and identity complexities often overlooked in conventional security discourses [5].

The human security paradigm is conceptualized through a seven-dimension index, which includes (1) community (in)security, including acts of terror, crime, identity conflicts or tension such as religious/ethnical/racial/sexual orientation; (2) economic (in)security, including persistent poverty and economic marginalization; (3) food (in)security, including famine, hunger, and increased food prices; (4) environmental (in)security, including environmental degradation and depletion of natural resources; (5) political (in)security, including human rights violations and political oppression; (6) personal (in)security, including acts of violence or human trafficking; and (7) health (in)security, including access to basic health care and addressing of epidemics [23]. This paradigm informed the study’s aims and research questions by guiding the exploration of how the seven dimensions impact the well-being of Somali women living in Kakuma Refugee Camp.

### Kakuma refugee camp

Kenya hosts more than 770,000 displaced persons of more than 23 nationalities. Many of these communities are located in United Nations High Commissioner for Refugees (UNHCR) camps, including Dadaab refugee complex in northeastern Kenya, one of the largest of its kind in the world, and Kakuma Refugee Camp and the adjacent Kalobeyei Settlement, both located in northwestern Kenya. Kakuma Refugee Camp comprises four sectors (Camps 1–4) and hosts approximately 288,206 humanitarian migrants from Somalia, South Sudan, Ethiopia, and the Democratic Republic of the Congo [6]. This remote camp is home to approximately 40,000 individuals of Somali descent and has hosted them for 30 years.

The infrastructure at Kakuma refugee camp faces significant challenges, including limited access to essential resources including inadequate shelter, poor transportation networks, and food insecurity. Poor transportation infrastructure limits mobility and access to essential services. Housing is often in a state of disrepair, reflecting the challenges of protracted displacement, prompting residents to modify their shelters to meet evolving family needs and support small-scale businesses [24,25].

While the UNHCR plays a critical role in the management and administration of the camp, the camp’s security falls under the jurisdiction of Kenya’s laws, with national security organizations charged with guaranteeing security [26]. The region’s main police station is located within Kakuma town, serving Kakuma Refugee Camp and the Kalobeyei settlement scheme, and is supported by police posts situated within the camps. These security facilities, comprising both the police station and police posts and patrol bases, form the backbone of the law enforcement infrastructure in the area. Security measures are implemented through a regime of spatial control, which includes road checkpoints, patrols, and armed raids. These measures, while aimed at maintaining public safety and security, protecting lives and property, and preventing criminal activities, have raised concerns about the humanitarian implications for the affected refugee communities [27]. Studies have confirmed widespread dissatisfaction with the performance of the National Police Service, with the foremost challenge being impeded access to criminal justice, identified as corruption [28].

In humanitarian crises, short-term refugee encampments are the ideal situation. They provide immediate relief in emergencies, ensuring access to basic necessities like food, water, healthcare, and shelter. They help manage the sudden influx of refugees, preventing strain on local resources and infrastructure, and serve as a temporary solution while longer-term arrangements, such as resettlement or integration, are organized [29]. Despite this, it is estimated that 78% of refugees around the world have resided in camps such as Kakuma for more than 5 years [30]. Refugees in long-term encampments face significant challenges, including limited mobility and employment opportunities. Further, the lack of sustainable solutions and reliance on external aid creates prolonged uncertainty. The health and mental health impact of long-term stays in humanitarian camps is significant. Confinement to camps exacerbates social isolation and negatively impacts mental health, leading to a cycle of dependency and marginalization [7,31,32].

To address the challenges of long-term encampment, Kakuma is in a transition phase, moving from encampment to an integration model with the aim of integrating residents into local governance structures. To this end, the Kenyan government recently instituted the Shirika Plan (formally the Marshall Plan), which is geared toward refugee self-sufficiency through setting up structures to provide essential services, including education, nutrition, health, security, and environmental conservation [33].

There are notable gaps in the literature on violence in humanitarian migration, particularly the limited attention to the intersectional vulnerabilities of women in long-term encampment. Unlike conventional security frameworks, the human security paradigm offers a strengths-based approach that moves beyond state-centric concerns to address interconnected, humancentric dimensions—making it well-suited to guide this study’s exploration of violence against women in protracted displacement. This present study answered the question: What factors are associated with experiences of violence among Somali women following migration to long-term encampment in Kakuma Refugee Camp?

## Methods

To address this question, the study employed a methodology informed by constructs of the human security paradigm. To integrate a gendered perspective, the study employed inclusive language that was sensitive to women’s lived experiences in the camp and attentive to their intersectional identities. Convenience sampling methods were used to enroll women of Somali origin (*N* = 260) between January and February 2024. This sample size meets recommended thresholds for structural equation modelling using WLSMV estimation. As Bowen & Guo note [34], samples of 200 or more are generally sufficient for models of moderate complexity, ensuring stable parameter estimates and reliable model fit. Focus on the Somali community was intended to allow for a more nuanced understanding of the community’s specific sociocultural dynamics, which might be obscured in a study targeting a broader audience. This targeted approach allowed the research team to delve deeper into the unique challenges and strengths that shape the experiences of Somali women in long-term encampment. Women were recruited from two villages (Kakuma 1 and Kakuma 3), which have a high number of Somali families.

Data was collected by female community health promoters who spoke two Somali dialects. The use of female interviewers was intended to enhance the comfort and openness of the participants, particularly when discussing sensitive topics such as interpersonal violence. Further, because the team were all community health workers with a basic understanding of trauma-informed care, they were well placed to foster an empathetic and supportive environment and elicit more honest and detailed responses.

All participants provided verbal consent prior to participation. The consent procedures were approved by the Kenyatta National Hospital–University of Nairobi (KNH-UON) Ethics and Research Committee and were documented and witnessed by trained data collectors. Verbal consent was preferred to accommodate participants with literacy challenges.

On average, each interview took 45–60 minutes. The instrument was linguistically and culturally validated and included measures of mental health, physical health, economic functioning, belonging, and service utilization. The study was reviewed by the Ethics and Research Committee and received research approval from the National Commission for Science, Technology and Innovation and the Department of Refugee Services, Kenya. For information on study methods, see the final research report [35].

### Dependent variable

The dependent variable “*are you experiencing any violence, exploitation, or abuse*” was a nominal variable (1 = *yes*, 0 = *no*). Women who responded in the affirmative were also asked to provide details on the types of violence they had recently experienced in their lives.

### Independent variables

The selection of independent variables was guided by the human security paradigm, which emphasizes freedom from fear and want across interconnected domains. Variables including age, marital status, food insecurity, health and mental health, attitudes toward intimate partner violence, and access to police and immigration services reflect these dimensions and help illuminate how structural and individual vulnerabilities intersect to shape women’s experiences of violence in long-term encampment. No additional variables were tested or excluded, as the selected covariates were deemed sufficient for the scope of the study. Variables were categorized as individual-level factors (age, education, marital status) and socioculture (attitudes toward IPV); economic functioning (household income, food security); health factors (physical and mental health functioning); and community-level factors (access to security institutions). Women’s empowerment was measured by women’s attitudes toward intimate partner violence [36]. The six questions asked of all women (irrespective of marital status) inquired whether it was justified for a husband to beat his wife if she, for instance, neglected children, burned food, argued, went out without the husband’s permission, used contraceptives, or denied the husband sex [37]. Food security was a composite of three variables capturing the number of days in the last month household members had had to eat less-preferred food, borrow food, or reduce food intake due to lack of money to buy food.

Mental health was captured using the Refugee Health Screener, a 13-item instrument used to measure depression, anxiety, and post-traumatic stress disorder symptoms. This screener has strong psychometric properties [38]. Versions of the Refugee Health Screener have been adapted for use among several migrant groups, including those of African ancestry [39]. Physical health was measured on a 4-item Likert scale ranging from *Very bad* to *Very good*. Women were also asked about ease of access to institutions, including law enforcement and police and immigration services. Both were assessed using 4-item Likert scales with variables ranging from *Not a problem at all* to *Very serious problem.*

Data was cleaned and recoded in SPSS [40] and Mplus [41], used to build structural equation models. To account for the categorical nature of the observed variables, a polychoric correlation matrix and the means and variance-adjusted weighted least squares estimator were used [42]. Mplus uses a pairwise method of handling missing values when the WLSMV estimator is used, which allowed all cases with full data to be included in the analyses [42]. Model fit was assessed using the root mean square error of approximation (RMSEA), the comparative fit index (CFI), and the Tucker–Lewis index (TLI), with values higher than 0.95 on the CFI and TLI indicating good fit [34].

## Results

Table 1 illustrates participant demographic characteristics. Respondents’ age ranged from 18 to 67 years; the average respondent was 35 years old (*SD* = 10.7). The most recent resident had lived in the camp for 1 year while the longest resident had lived in the camp for 28 years (*M* = 14.1, *SD* = 3.8). Women lived in households with an average of 7 individuals and an average of 3.6 children (*SD* = 2.4). Slightly more than half the respondents did not have any formal education. Approximately 24.6% reported they had no education at all, and another 26.9% had attended only madrasas. Of the women who reported formal schooling, only 35.8% had received their education in Kenyan schools, with Somalia as the country of most education earned (39.2%). More than half of respondents were currently married (57.7%), with others self-identifying as divorced (20.8%), single, (15.8%), or widowed (5.8%). Of those who were currently married, approximately 11.5% were in polygamous unions. It is important to note that 6.9% of these respondents were unsure about the current plurality status of their unions. Most women in polygamous unions identified their ranking as either first or second wife.

**Table 1:** Demographic characteristics (*N* = 260).

Variable	N (%)
**Education**	
No education	64 (24.6%)
Madrasa only	70 (26.9%)
Some primary	73 (28.1%)
Secondary + college	50 (19.2%)
**Marital status**	
Married	150 (57.7%)
Single	41 (15.8%)
Divorced/separated	54 (20.8%)
Widowed	15 (5.8%)
**Health outcomes**	
Very good	70 (26.9%)
Good most of the time	116 (44.6%)
Not good	59 (22.7%)
Very bad	15 (5.8%)
**Variable**	***M* (*SD*) Range**
Age	35 (10.7) 18–67 years
Food security index	21.45(10.77) zero to 60
Household income	6,194 (5,851) zero to KES 50,000
Refugee Health Screener-13 mental health score	19.7 (13.7) zero to 52

Women reported multiple streams of income, with cash transfers from government and nongovernmental sources as the most cited source (*n* = 258), followed by transfers from family members (*n* = 216). The average household had a monthly income of KES 6,194 (approximately $50), with household incomes ranging from zero to KES 50,000. The income variable was square root transformed to account for a moderate positive skewness. Finally, approximately 78% of women reported having to borrow money to meet daily household needs.

All but six households were currently receiving food assistance, including in-kind food distributions. This finding is not surprising, given women’s status as humanitarian migrants entitled to nutritional assistance while in the camp. Women reported that this food assistance lasted only halfway through the month, highlighting the ongoing challenges they face in accessing adequate resources (*M* = 18.1, *SD* = 6.5). A food security index was constructed from three food consumption variables ranging from 0 to 60 (*M* = 21.4, *SD* = 10.7), with households with higher scores experiencing greater food insecurity. On average, households reported that they went 8.5 days eating less preferred foods, 7 days having to reduce the number of meals consumed, and 5.5 days borrowing money to meet household food needs due to lack of food or money to buy food.

Women were asked to rate their overall physical health on a 4-point scale. Due to small cell sizes, the health variable was collapsed into two categories, with 71.5% of respondents rating their health as *Good* or *Very good*. The 13-item Refugee Health Screener had good internal consistency (α = 0.9) and was dichotomized using the suggested cutoff score of 11 points, to indicate mental health issues. Approximately 34.2% of all respondents screened positive for mental ill-health.

Women had progressive attitudes toward domestic violence. For instance, 80.8% indicated that beating a wife who goes out without telling her husband is not acceptable, and 73.5% stated that it is unacceptable to beat a wife who denies her husband sex. Women’s attitudes toward intimate partner violence had strong reliability (α = 0.83) with a one-factor solution confirmed by fit statistics and parameter estimates [*χ*²(9, *N* = 260) = 16.57 value, *p* = 0.055, RMSEA = 0.057 (90% CI 0.000–0.09), CFI = 0.95, TLI = 0.99]. Women were asked about access to safety institutions, particularly the police and immigration services (see Table 2). Approximately 19% of women said that accessing police services was somewhat a problem, a serious problem, or a very serious problem. In addition, 21.1% of respondents indicated that access to immigration services was somewhat a problem, a serious problem, or a very serious problem for them. Finally, approximately 31.2% of the women reported experiencing abuse, with the most cited categories being abuse/insults (*n* = 74), physical abuse (*n* = 44), and threats (*n* = 34).

**Table 2:** Experiences of violence (*N* = 260).

Variable	*N* (%)
**Experiences of abuse**	
Yes	81 (31.2%)
No	179 (68.8%)
**Access to police**	
Not a problem	187 (71.9%)
Somewhat a problem	21 (8.1%)
Serious problem	13 (5.0%)
Very serious problem	11 (4.2%)
Missing	28 (10.8%)
**Access to immigration officers**	
Not a problem	181 (69.6%)
Somewhat a problem	22 (8.5%)
Serious problem	6 (2.3%)
Very serious problem	21 (8.1%)
Missing	30 (11.5%)

Structural equation modeling (SEM) was used to determine the factors associated with experiences of violence. To address missing data on the categorical variables —access to police and immigration services—multiple imputation in Mplus was used to generate 52 datasets with a categorical imputation model which were pooled for analysis using Rubin’s rules [43]. The final SEM model had adequate fit [*χ*2 (68) = 80.2, *p* ≤ 0.147, RMSEA = 0.029 (90% CI 0.000–0.051), CFI = 0.98, TLI = 0.98]. Individual level factors—marital status (*B* = 0.49), age (*B* = 0.02), and household food security (*B* = 0.02)—were associated with experiences of violence. Further, mental health (*B* = 1.34), attitudes towards IPV (*B* = 0.23), and access to police (*B* = 1.17) and immigration services (*B* = 1.05), each increased the probability of violence.

Older women (*B* = 0.02, *p* = 0.04), those who were currently married (*B* = 0.49, *p* = 0.03), and those living in a food-insecure household (*B* = 0.02, *p* = 0.04) were more likely to experience violence. From a health security perspective, a positive score on the Refugee Health Screener–13 was significantly associated with experiences of violence (B = 1.34, p = < .001). From a sociopolitical security perspective, permissive attitudes toward intimate partner violence were linked to increased likelihood of experiencing violence (B = 0.23, p = .03). Additionally, women who encountered barriers when seeking assistance from police (B = 1.17, p = .013) or immigration services (B = 1.05, p = .021) were more likely to report experiences of violence. Supplementary S1 and S2 Tables report factor loadings and residual variances for latent construct attitudes of IPV, followed by full SEM results including all paths—significant and non-significant—with Unstandardized B, SE, 95% CI, p-values, and Standardized β.

## Discussion

The integration of gender perspectives into the human security paradigm has been a subject of extensive scholarly critique [4]. The human security paradigm’s versatility and emphasis on the structural and individual root causes of insecurity (Hudson, 2005) allows for a deeper analysis of how situations such as long-term encampment affect women. The liminality of migration poses significant challenges, including precarity for the migrant, and women’s vulnerabilities are exacerbated [44]. This study aligns with past research indicating that women’s experiences of violence sit at the intersection of individual factors, formal institutions (security infrastructure, laws, and social services), and informal institutions (sociocultural norms) [45]. Addressing an issue as complex as violence in long-term migrant encampments demands a transformative approach [46] that recognizes the intersectionality of gender and other forms of identity, including motherhood, economic status, and immigration status. Further, it requires an approach that pays attention to the systemic causes of violence against women.

These data indicate that food insecurity increases the likelihood of women experiencing violence. Food insecurity often leads to heightened household stress, which can exacerbate gender inequalities. Women in food-insecure households are more vulnerable to violence as they may be unable to meet expected gender roles. Additionally, other members of these households may perpetrate violence due to the frustration associated with food scarcity. Several food assistance programs exist in Kakuma Refugee Camp, including Bamba Chakula, which provides electronic cash transfers to be used for food purchases. The program is designed to increase access to a variety of foods and provide more autonomy over dietary choices [47]. However, fluctuating funding and food ration cuts undermine the camp’s food security. To proactively mitigate the negative effects of food insecurity, broader interagency collaborations and diversifying nutrition interventions to complement existing programs should be considered.

Culture plays a significant role in shaping how individuals perceive and address violence. In the context of sub-Saharan Africa, cultural norms propagate subjugation, decreasing the autonomy and agency of women. Norms and mores that stymie women’s autonomy, particularly bodily autonomy, increase their vulnerabilities to violence. And while it is not a panacea for violence, supporting women’s autonomy is integral even as we address institutional barriers to well-being. These findings demand autonomy over all facets of women’s lives, including income-generating opportunities, food security, and mental well-being. International and nongovernmental organizations offering gender-based violence prevention and detection programs, including the UNHCR and the International Rescue Committee [48,49], should adopt a transformative approach that supports women’s agency while addressing structural impediments to well-being.

In societies where violence is normalized due to histories of war [13], patriarchal norms that justify violence against women [50], or the glorification of revenge tactics [51], victims may shy away from accessing legal mechanisms. This is mainly due to pressure from their communities and fear of ostracism. In the present study, approximately 72% of respondents reported that law enforcement is accessible to them. However, when women are disconnected from law enforcement, they are unable to access or help inform investigative procedures. For instance, despite police presence within the camp, data points to a gap between known gender cases of violence and those reported to police [48]. Refugee camps like Kakuma, intended as safe havens for displaced people, often end up acting as long-term placements and are plagued by various forms of violence. Indeed, media reports that continued tensions have escalated into fatalities [52,53] within the Kakuma camp.

### Implications

Individual-level implications of this study underscore the importance of prioritizing women’s agency by supporting autonomy across all aspects of life, including bodily autonomy, income generation, and decision-making. Effective interventions must account for the intersectionality of identities that compound women’s vulnerability to violence. To counter restrictive cultural norms, programs should include norm-challenging components that empower women and foster greater independence.

Structural-level implications highlight the need strengthen community-based accountability and protection systems is essential to bridge gaps in formal reporting and enforcement mechanisms. Programs must acknowledge that camps like Kakuma function as complex, long-term living spaces and therefore require sustained investment in safety and support infrastructure. Finally, both national and international actors must commit to structural reforms that center the voices of survivors and confront the institutional inequalities that perpetuate violence.

### Strengths and limitations

This paper does not aim to address the lived experiences of male survivors of violence, or to imply that the atrocities committed against women are more noteworthy than those perpetrated against men. Neither does it seek to efface the valiant efforts of international organizations in combating atrocities perpetrated during war and conflict. Rather, it seeks to emphasize why bringing gender from the footnotes of the conversation to the main text would be focal in the creation of gender-responsive solutions for both genders. Although our study is not free from methodological limitations, it highlights the voices of Somali women who have endured life in Kakuma Refugee Camp in Kenya.

The cross-sectional design of this study limits the ability to infer causality or track changes over time. Therefore, future research should more closely examine migration out of violence, violence experienced during migration, and migration into violent contexts—phenomena that align with what conflict scholars have described as the “continuum of violence in conflict” [54,55].

While recruitment efforts were robust, this was a convenience sample, meaning that results are biased toward the women who felt comfortable participating in the study and do not represent the broader population. Future research should consider longitudinal designs and random sampling to improve the robustness and generalizability of the findings. Further, given the narrow scope, only Somali women were recruited into the study; therefore, the study does not encompass the diversity of women living in the camp. This study faced missing data primarily in response to questions on topics that are particularly sensitive for refugee women in Kakuma. Despite efforts to ensure anonymity, non-response likely stemmed from fear, stigma, or concerns about safety and confidentiality [56]. These challenges are common in conflict-affected settings and may limit the generalizability of some findings. Future research could explore alternative, trust-building methods to better capture these experiences ethically. Finally, while Kenya offers asylum to individuals in need of protection due to their sexual orientation or gender identity, this study did not explore the experiences of transgender women.

## Conclusion

This study underscores the need for targeted interventions and support systems that address the unique challenges faced by women in long-term encampment. The findings reveal a complex interplay of factors that exacerbate the risks of violence. The study’s exclusive focus on Somali refugees is both relevant and necessary, given the unique challenges and experiences faced by each specific community in the camp. This targeted approach contributes to a more nuanced understanding of Somali women’s experiences, which may differ significantly from people from other countries. By amplifying the voices of refugee women and prioritizing their needs, we can foster resilience and create safer environments.

## Supporting information

S1 Table Exploratory Factor Analysis (EFA): Attitudes toward intimate partner violence. (DOCX)

S2 Table Factors associated with experiences of violence: Structural equation modeling results. (DOCX)

S1 Checklist Inclusivity global research questionnaire. (DOCX)
