# Supplementary material for: Using a human security lens to examine experiences of violence against women in long-term encampment
Source: PLoS One. 2025 Nov 6;20(11):e0336028. doi: 10.1371/journal.pone.0336028 (PMC12591481; doi:10.1371/journal.pone.0336028)
Supplement: S2 Table — (DOCX) [file pone.0336028.s002.docx]

**Using a Human Security Lens to Examine** **Experiences of Violence Against Women in Long-Term Encampment**

**S2 Table: Factors Associated with Experiences of Violence: Structural Equation Modeling Results**

| **Unstandardized Estimates B** | | | | |
| --- | --- | --- | --- | --- |
| **Predictor** | **Estimate** | **SE** | **z-value** | **p-value** |
| IPV attitude (latent) | 0.233 | 0.113 | 2.073 | 0.038 |
| Food security index | 0.020 | 0.010 | 1.995 | 0.046 |
| Mental health | 1.340 | 0.265 | 5.061 | 0.000 |
| Martial status | 0.493 | 0.236 | 2.089 | 0.037 |
| Household income (SQRT) | 0.003 | 0.003 | 0.979 | 0.328 |
| Education status | 0.454 | 0.250 | 1.817 | 0.069 |
| Age | 0.025 | 0.012 | 2.054 | 0.040 |
| Health status | -0.117 | 0.243 | -0.481 | 0.631 |
| Access to police | 1.175 | 0.471 | 2.496 | 0.013 |
| Access to migration support | 1.054 | 0.458 | 2.300 | 0.021 |
| **Standardized Estimates β** | | | | |
| **Predictor** | **Estimate** | **SE** | **z-value** | **p-value** |
| IPV attitude (latent) | 0.140 | 0.066 | 2.117 | 0.034 |
| Food security index | 0.152 | 0.074 | 2.058 | 0.040 |
| Mental health | 0.443 | 0.075 | 5.937 | < .001 |
| Martial status | 0.164 | 0.077 | 2.144 | 0.032 |
| Household income (SQRT) | 0.088 | 0.088 | 1.001 | 0.317 |
| Education status | 0.155 | 0.086 | 1.790 | 0.073 |
| Age | 0.182 | 0.086 | 2.109 | 0.035 |
| Health status | -0.036 | 0.076 | -0.480 | 0.631 |
| Access to police | 0.249 | 0.094 | 2.638 | 0.008 |
| Access to migration support | 0.235 | 0.099 | 2.364 | 0.018 |

*χ*2 (68) = 80.2, *p* ≤ 0.147, RMSEA = 0.029 (90% CI 0.000–0.051), CFI = 0.98, TLI = 0.98.
